# An efficient low-shot class-agnostic counting framework with hybrid encoder and iterative exemplar feature learning

**DOI:** 10.1371/journal.pone.0322360

**Published:** 2025-06-06

**Authors:** Qinghua Yang, Bin Liu, Yan Tian, Yangming Shi, Xinxin Du, Fangyuan He, Jikun Guo

**Affiliations:** 1 School of Artificial Intelligence, China University of Mining and Technology (Beijing), Beijing, China; 2 Energy Ningxia Coal Industry Co., Ltd., YangChangWan Coal Mine, YingChuan, NingXia, China; 3 College of Applied Science and Technology of Beijing Union University, Beijing, China; 4 School of Electronic and Information Engineering, Guangdong University of Petrochemical Technology, Guangdong, China; Khalifa University of Science and Technology, UNITED ARAB EMIRATES

## Abstract

Few-shot learning techniques have enabled the rapid adaptation of a general AI model to various tasks using limited data. In this study, we focus on class-agnostic low-shot object counting, a challenging problem that aims to achieve accurate object counting with only a few annotated samples (few-shot) or even in the absence of any annotated data (zero-shot). In existing methods, the primary focus is often on enhancing performance, while relatively little attention is given to inference time—an equally critical factor in many practical applications. We propose a model that achieves real-time inference without compromising performance. Specifically, we design a multi-scale hybrid encoder to enhance feature representation and optimize computational efficiency. This encoder applies self-attention exclusively to high-level features and cross-scale fusion modules to integrate adjacent features, reducing training costs. Additionally, we introduce a learnable shape embedding and an iterative exemplar feature learning module, that progressively enriches exemplar features with class-level characteristics by learning from similar objects within the image, which are essential for improving subsequent matching performance. Extensive experiments on the FSC147, Val-COCO, Test-COCO, CARPK, and ShanghaiTech datasets demonstrate our model’s effectiveness and generalizability compared to state-of-the-art methods.

## Introduction

Deep learning technologies has become an essential component across various fields [1, 2]. To tackle the challenges posed by dynamic changes in task diversity, few-shot learning [3, 4] facilitates rapid model adaptation with limited annotated data, significantly enhancing the flexibility and operational efficiency of systems. Moreover, some applications extend beyond object recognition, requiring quantitative or density-based analyzes to fulfill specific task requirements. In this paper, we focus on low-shot object counting (LSC), which encompasses both few-shot counting (FSC) and Zero-Shot Counting (ZSC).

Object counting aims to count objects of interest within an image. Early studies primarily focused on counting specific categories, such as crowds [5–7], vehicles [8, 9], animal species [10–12], and plants [13, 14]. However, these methods typically demand extensive annotated datasets for training and exhibit limited adaptability for counting objects in novel categories, thus limiting their applications.

To overcome these limitations, GMN [15] proposed class-agnostic counting (CAC), which aims to count any class of object based on given exemplars. Following the release of the challenging FSC147 dataset [16], few-shot object counting (FSC) has garnered significant attention in the research community [17–31], aiming to enable counting in arbitrary categories using only a few exemplars. This advancement enables models to generalize to unseen classes, offering promising applications across diverse scenarios.

**Fig 1:**
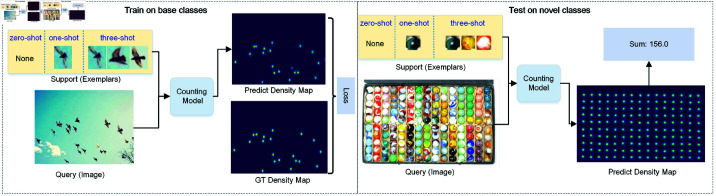
Illustration of low-shot object counting, which includes few-shot (1-shot or 3-shot) object counting, where a few support exemplars are provided, and zero-shot object counting, which counts the most frequent class objects.

As illustrated in Fig 1, in LSC, the model is trained on base classes using only a few (or none) exemplars and tested on novel classes under low-shot settings. ZSC is a reference-less, class-agnostic approach aimed at counting the most frequently occurring class in an image.

Most current FSC methods follow an extract-then-match paradigm: first extracting features from both the image and exemplars, processing them, and then matching them by calculating the similarity between the query image and exemplar features. The resulting similarity map is then regressed to generate a density map, with the sum of the density map representing the object count. These methods primarily differ in how image embeddings and exemplar-feature similarities are constructed. ZSC methods follow a similar paradigm but rely on identifying exemplars based on repeated objects in the image [18] or via attention mechanisms [19].

However, as model performance improves, these methods have become increasingly complex, leading to higher training costs and slower inference speeds. Real-time performance is critical for practical applications, particularly in UAV operations. However, existing CAC counting methods seldom address or thoroughly analyze this aspect.

We analyze existing low-shot counting methods, comparing their model sizes and inference speeds. Through our analysis, we find that limitations in the Transformer attention modules’ ability to process image feature maps often lead to high training costs, slow convergence, and restricted feature spatial resolution. To overcome these issues, we propose a hybrid encoder that reduces training costs and inference time. Specifically, we use a hybrid encoder to extract multi-scale features from the backbone, which is critical for accommodating various object sizes. Multi-head self-attention (MHA) is applied only to top-level image features, which are reduced to $\frac{1}{32}$ of the original image size. Given that computational complexity increases sharply with feature size, this strategy reduces computational cost significantly compared to vanilla attention applied at multiple levels (e.g., $\frac{1}{8},\frac{1}{16},\frac{1}{32}$). We then employ a two-path, top-down and bottom-up approach using convolutional neural netowrks (CNN) to fuse features across different levels.

To ensure accurate exemplar matching, we iteratively update the exemplar features by learning from query image features. First, we extract shape information from the exemplar, project it to a high-dimensional space, and fuse exemplar features with the shape embeddings via cross-attention. The fused exemplar features then interact with the image features via cross-attention, learning from other similar objects in the image to enrich the exemplar features with class-level characteristics, thus improving followed matching accuracy.

To address overlap issues in the ground truth (GT) density map, which arise from considering only the shape size of a few-shot exemplar in an image, we propose a method to dynamically generate a more accurate GT density map by combining both object shape and distance information among GT points in the image, providing more precise supervisory information during training.

Our contributions can be summarized as follows:

- we propose a method to adaptively generate a more accurate GT density map by combining both object shape and distance information among GT points in the image.

- We propose a novel low-shot counting model incorporating an effective hybrid encoder, which achieves real-time inference without compromising high performance.

- Experiments on counting benchmarks demonstrate the effectiveness of our approach. Compared to state-of-the-art density-based method [24], our model achieved substantially lower latency (18.22 ms vs. 44.77 ms on the validation set, 17.92 ms vs. 45.37 ms on the test set) and reduced MAE by 9.5% and 6.3% on the validation and test sets, respectively.

## Related works

Early object counting tasks were initially approached with class-specific detectors, which could accurately locate object positions. However, these detection-based methods struggle in occluded and crowded scenarios. To address this limitation, regression-based methods [10, 32, 33] were developed, treating counting as a supervised regression task. Under this paradigm, most research focuses on optimizing model architectures [33], multi-scale strategies [34, 35], or novel learning targets [32, 36]. These approaches only require point annotations, which are less labor-intensive than detection-based methods that rely on bounding box annotations. However, all of these methods require large training datasets and are limited to specific classes, making them less generalizable to unseen classes.

Few-shot counting (FSC) has gained significant attention due to its ability to count objects using only a few exemplars as references, with adaptability to novel classes during testing. GMN [15] introduced a generic matching network architecture for class-agnostic counting, extracting exemplar and image features in a two-stream fashion, pooling the exemplar features into 1 × 1 dimensions, and concatenating with the query features for regression into a density map. To address the unreliable location precision caused by direct concatenation, CFOCNet [17] drew on Siamese network principles from object tracking [37], using the exemplar feature as a 2D kernel to convolve over the query feature map to compute similarity. FamNet [16] introduced the widely used FSC147 dataset for FSC research and proposed a Siamese backbone adaptation strategy to improve correlation robustness during testing. BMNet [21] proposed a similarity-aware framework that jointly learns representations and similarity metrics end-to-end, using self-attention to reduce intra-class appearance variability in the test image. SAFECount [23] introduced a similarity-aware feature enhancement block that compares exemplar and query image features to create a score map, subsequently generating a reliable similarity map. This is followed by a feature enhancement module that uses similarity values as weighting coefficients to integrate the support features into the query image features. CounTR [22] proposed a Transformer-based architecture [38] that uses cross-attention to fuse image and exemplar features and employs a two-stage training regimen, starting with self-supervised pre-training and followed by supervised fine-tuning. LOCA [24] separately considers exemplar shape and appearance features, iteratively adapting them into object prototypes, while DAVE [30] implements a detect-and-verify paradigm that generates a high-recall detection set and verifies detections to filter out outliers.

More recently, with the advancement of large language models, some studies have explored open-world object counting, integrating both visual and language modality features. In this paradigm, objects of interest can be specified by text (class names or descriptions), exemplars, or both. ZSC [26], CLIP-Count [25], and VLCounter [28] target zero-shot object counting, while CounTX [27] can directly predict object counts using an inference image and an arbitrary object class description. Although these methods do not yet perform as well as previous approaches, they show considerable potential.

Our approach falls within density-based methods, using state-of-the-art LOCA [24] as our baseline for comparison.

## Methods

### Preliminaries

We adopted the general settings of few-shot counting (FSC), training our model on base classes ${𝒞}_{b}$ and evaluating it on novel classes ${𝒞}_{n}$, where there is no overlap between the two, i.e., ${𝒞}_{b}$ ∩ ${𝒞}_{n}=\emptyset$. The ground truth annotations for each image comprise the center points of all objects within the target classes, along with the bounding boxes of *K* (K-shot) representative exemplars. Generate the ground truth density map based on the points. Given a query image $I\in {\mathbb{R}}^{H_{0}\times W_{0}\times 3}$, the counting model predicts a density map $\hat{P}\in {\mathbb{R}}^{H\times W}$. Summing the values in $\hat{P}$ yields the estimated object count in *I* for the specified class.

### Model architecture

**Fig 2:**
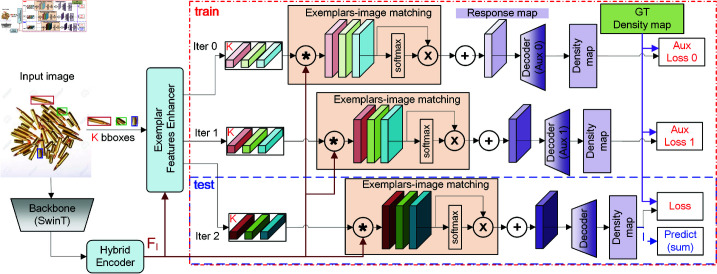
The architecture of our model. Hybrid Encoder is a module to enhance image features. i-EFL (iterative Exemplar Feature Learning) module is used to iteratively enriching exemplar features from image. The symbol ‘*’ is a convolution with enhanced exemplar features as kernel. ‘x’ is multiply the weights after softmax. ‘+’ is to generate a response map.

As shown in Fig 2, our model architechture proceeds through five modules: (i) image feature extraction (backbone), (ii) image feature enhancement (hybrid encoder), (iii) exemplars feature learning (iteratively update exemplar feature), (iv) exemplar-image matching (similarity maps), (v) density regression(decoder, generate density map).

The input image is resized to *H* × *W* pixels, and image augmentation techniques, including tiling, color jittering and horizontal flipping [22]. Multi-scale image features are extracted using a Swin Transformer [39] backbone. To reduce the computational cost of attention mechanisms, our hybrid encoder applies attention only to high-level features, complemented by cross-scale fusion with CNN layers. Then the high level features ($S_{5},S_{4}$) are upsampled to the same size. Concatenated different level features and projected to generate the encoded image features $F_{I}\in {\mathbb{R}}^{h\times w\times d}$.

Next, for the *K*-shot exemplars, RoIAlign is used to obtain exemplar features. In the iterative exemplar feature learning (i-EFL) module, a learnable shape embedding is fused with exemplar features using multi-head cross-attention (MHCA) and then enriched with image features via another MHCA, producing exemplar features with class-level commonalities.

In the matching module, image features are depth-wise correlated with the enhanced exemplar features, generating a similarity tensor. The similarity tensors of the exemplars are reweighted by their softmax scores and combined to produce a joint response tensor (Response Map).

Finally, a progressive up-sampling regression head in the decoder module predicts the final density map, where the sum of its values represents the total count of objects in the image relevant to the exemplars.

### Ground truth density map generation

**Fig 3:**
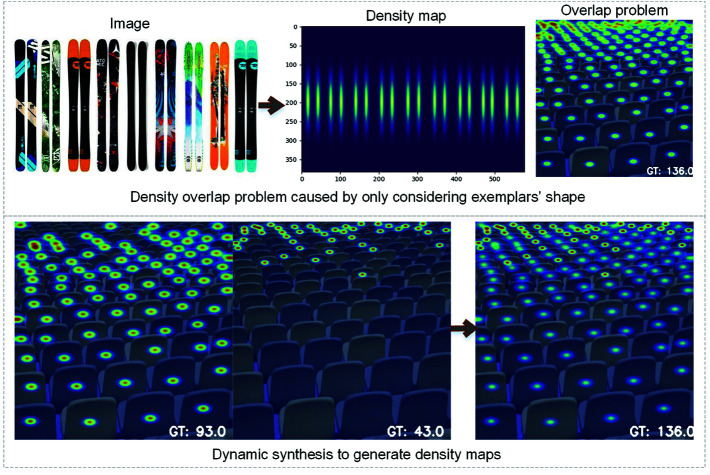
Consider shape information to generate density map, but confronts overlap problem when the target depth varies greatly. Adaptively adjust the window size based on point distances to solve the above issue.

The density map is essential in density-based counting models. To more effectively capture shape features, we adaptively generate the ground truth density map based on object size and inter-point distances. Specifically, we calculate the average height $\bar{h}=\frac{1}{K}\sum h_{i},i\in \{1,\cdots ,K\}$ and width $\bar{w}=\frac{1}{K}\sum w_{i},i\in \{1,\cdots ,K\}$ of objects from the *K*-shot annotated boxes, which decide the window size of the Gaussian function used to generate the density map. As illustrated in the left image of Fig 3, the density map corresponding to a object accounts for both its length and width, rather than merely generating a circular region. However, when objects vary widely in size and distribution density, as shown in the right image of Fig 3, densely packed regions in the generated density map may suffer from significant overlap, leading to inaccuracies. To address this, we use a hybrid density generation method that accounts for both object shape and neighbor distances. For each point, we compute the distance to all other points, taking the average of the three smallest values as its distance metric *d*. Based on this metric, the window size is dynamically adjusted, and a Gaussian density map $D_{\text{map}}$ is generated. Points in the image are grouped by the value of *d*, and our experiments indicate that two groups are sufficient for accurate density map generation. The steps are as follows:

$$mask=(\alpha min(\bar{h},\bar{w})<=d)$$ (1)

$$D_{1}(points[mask])=\text{Gaussian}(points[mask],\beta_{1}(\bar{h},\bar{w}))$$ (2)

$$D_{2}(points[\sim mask])=\text{Gaussian}(points[\sim mask],\beta_{2}(\bar{h},\bar{w}))$$ (3)

$$D_{\text{map}}=D_{1}+D_{2}$$ (4)

where the hyper parameter α is used to filter points by adjusting the ratio of object size to distance metric, while $\beta_{i}$ is used to adjust the parameter of window size in Gaussian filter, with $\beta_{2}<\beta_{1}$. As shown in the second line of Fig 3, the left image is generated using Eq (2), the middle image is generated using Eq (3). Combining these maps produces a final density map where the overlap problem is alleviated, resulting in a more accurate ground truth density map.

#### Hybrid encoder.

In the FSC147 dataset [16], object sizes vary significantly, making multi-scale features essential for improving counting accuracy, accelerating training convergence, and enhancing performance [40]. Despite the strong performance of Transformers, one key limitation is their high computational and memory requirements for large numbers of key elements.

**Fig 4:**
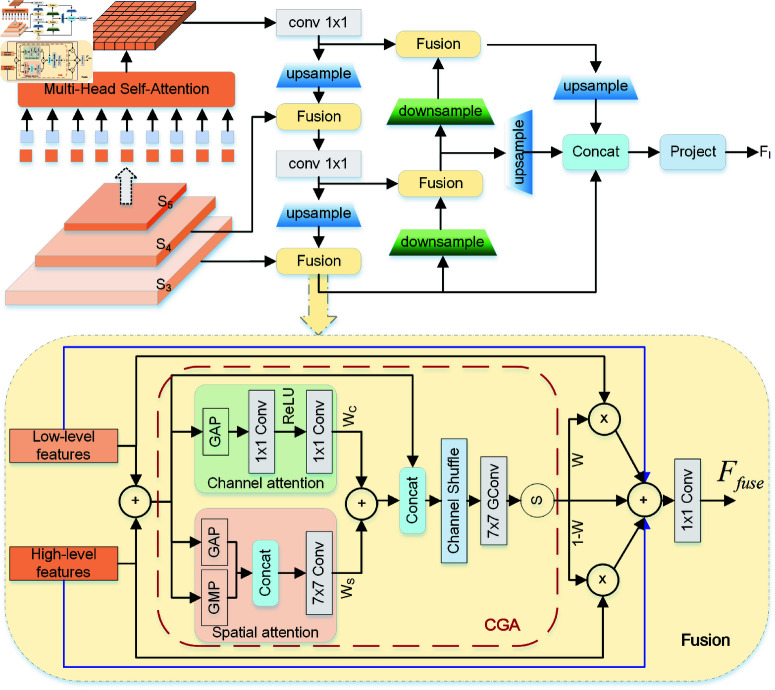
The structure of hybrid encoder. Composition: a High-Level Features Self-Attention (HFSA) mechanism and a Cross-Level Features Fusion (CFF) module. The figure below shows the detailed structure of the CFF module.

To address this, we introduce a hybrid encoder module, combining High-level Features Self-Attention (HFSA) and Cross-level Features Fusion (CFF). The encoder structure is shown in Fig 4. High-level features (S5) occupy only $\frac{1}{64}$ of the input image, yet contain richer semantic information, capturing relations among conceptual entities, thereby aiding object localization and recognition. As analyzed in RT-DETR [51], applying self-attention to high-level features captures conceptual connections, enhancing subsequent modules’ ability to recognize objects. Lower-level intra-scale interactions, however, are unnecessary due to the lack of semantic concepts and the risk of redundancy and confusion with high-level feature interactions.

In the CFF module, two adjacent scale features are fused via top-down and bottom-up pathways. Finally, we resize all feature levels to a consistent size, concatenate them, and project to a lower dimension, yielding the final image features *F*_*I*_. This process is described by:

$$F_{5}=MHA(S_{5},S_{5},S_{5})\;F_{I}=CFF(S_{3},S_{4},F_{5})$$ (5)

In the CFF module, we employ a content-guided attention (CGA)-based mixup fusion scheme [49], which effectively fuses features and enhances gradient flow. As shown in Fig 4, CGA generates channel-specific spatial importance maps (SIMs), producing an exclusive SIM for each input channel in a coarse-to-fine manner. This approach integrates both channel and spatial attention weights, ensuring effective information interaction and guiding the model to focus on significant regions within each channel.

#### Iterative exemplar features learning.

**Fig 5:**
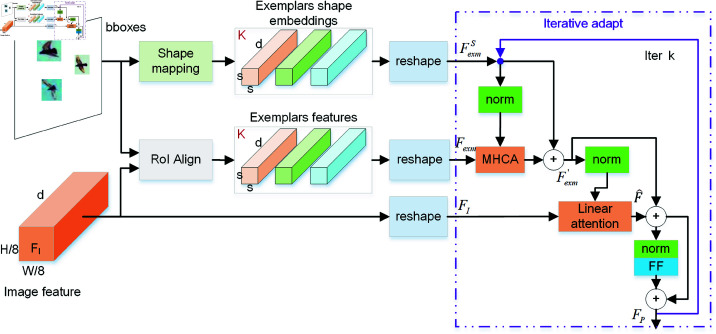
Iterative exemplar features learning module.

The structure of the iterative exemplar feature learning module is illustrated in Fig 5, which is almost the same to the module of object prototype extraction in LOCA [24], and we elaborate on its details below.

In the *K*-shot setting, given *K* bounding boxes $\{b_{i}{\}}_{i\in \{1:K\}}$ as support exemplars, we first obtain *K* exemplar features $f_{exm}\in {\mathbb{R}}^{s\times s\times d}$ via RoIAlign [41]. To learn the shape information of each exemplar, we apply a mapping function Φ(·) that maps each object’s height and width $\left[h_{i},w_{i}]\in {\mathbb{R}}^{2},i\in \{1:K\right\}$ to match the shape of the exemplar features ${\mathbb{R}}^{s\times s\times d}$. This function is implemented as a multilayer perceptron (MLP) with three linear layers, each followed by ReLU activations. After mapping, we obtain the shape embeddings $f_{exm}^{S}$.

Next, we fuse the exemplar features and shape embeddings using multi-head cross-attention to generate refined exemplar features $F_{exm}^{\prime }$. These features are further fused with the image feature *F*_*I*_ through cross-attention, querying exemplar similar objects in the image to learn class-level characteristics and generate prototype-like exemplar features *F*_*P*_. To reduce computational complexity, we have replaced the cross attention with linear attention here. To acquire more generalized exemplar features, we iteratively update *F*_*P*_ by repeating the above steps. Since the query image contains multiple objects of the same class as the exemplar, this iterative approach enables the model to capture additional similarities within the same category, yielding generalized exemplar features. The iterative process of the algorithm as follows:

**Algorithm 1: Iterative to update the exemplar features.**

Input:

  Exemplar shape features: $F_{exm}^{S_{0}}=MLP([h_{i},w_{i}]),\;i\in \{1:K\}$

  Exemplar features: *F*_*exm*_

  Image features: *F*_*I*_

Iterative update

  for the iter k:

  1. $F_{exm}^{\prime k}=MHCA(F_{exm}^{S_{k}},F_{exm},F_{exm})+F_{exm}^{S_{k}}$

  2. ${\hat{F}}^{k}=MHCA(F_{exm}^{\prime k},F_{I},F_{I})+F_{exm}^{\prime k}$

  3. $F_{exm}^{S_{k+1}}=FF({\hat{F}}^{k})+{\hat{F}}^{k}$

  4. Then repeat step 1.

where MHCA denotes multi-head cross attention. In our experiments, we performed two iterations, resulting in three generalized exemplar features $F_{P}^{1},F_{P}^{2},F_{P}^{3}$ for subsequent matching operations. During training, $F_{P}^{1}$, $F_{P}^{2}$ are used alongside $F_{P}^{3}$ to assist in model training. For testing, only the final feature $F_{P}^{3}$ is used for matching.

### Training losses

The training loss is defined as the Mean Squared Error (MSE or *l*_2_ loss) between the predicted and ground-truth density maps in pixel space:

$${ℒ}_{C}=\frac{1}{N_{p}}{\left\|\hat{P}-G\right\|}_{2}^{2}$$ (6)

where *N*_*p*_ represents the number of objects that calculated from G, $\hat{P}$ is the model’s predicted density map, and G is ground-truth density map.

Additionally, the intermediate enhanced exemplar features are matched with image features to generate auxiliary density maps ${\hat{P}}_{aux}^{i}$. The auxiliary loss is calculated as:

$${ℒ}_{aux}=\frac{1}{N_{p}}\sum_{k\in 1,2}\overset{2}{\underset{2}{\left\|{\hat{P}}_{aux}^{k}-G\right\|}}$$ (7)

Thus, the total loss is

$$ℒ={ℒ}_{C}+\lambda {ℒ}_{aux}$$ (8)

where the λ=0.3 in the paper.

## Experiments

### Datasets and metrics

#### FSC147.

The FSC-147 dataset [16] was introduced for few-shot object counting tasks and comprises 6,135 images across a wide range of 147 object categories, ranging from kitchen utensils and office stationery to vehicles and animals. The dataset exhibits a significant variation in object counts, with images containing between 7 and 3,731 objects and an average of 56 objects per image. In each image, every object instance is annotated with a dot marking its approximate center. Additionally, three object instances per image are randomly selected as exemplars, each accompanied by axis-aligned bounding box annotations.

**Fig 6:**
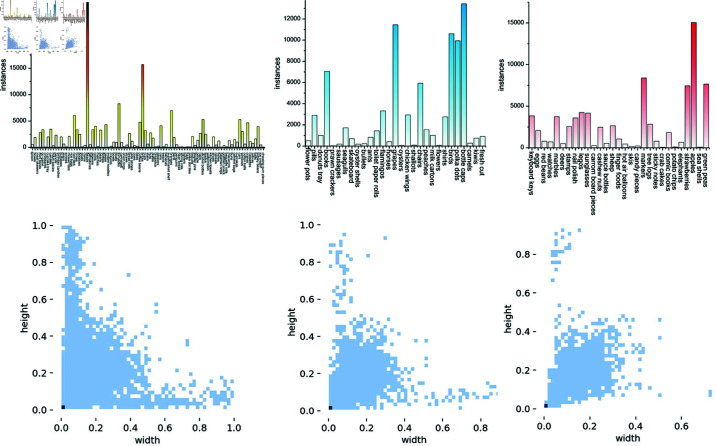
The first row of images illustrates the distribution of classes and the number of instances across the training, validation, and test sets, respectively. The second row visualizes the relationship between width and height across these datasets. The model is trained on 89 classes and evaluated on 29 classes for both validation and testing.

Following [16], we partitioned the dataset into 89 object categories for training, 29 for validation, and 29 for testing. These subsets comprise 3,659 images in the training set, 1,286 images in the validation set, and 1,190 images in the test set. Fig 6 illustrates the width and height distributions of objects across these sets. Notably, while many objects in FSC-147 are small, the dataset demonstrates a substantial range in object sizes.

#### CARPK & ShanghaiTech.

To further evaluate the generalizability of the model, we introduce two datasets designed for specific class counting. The CARPK dataset [8] is a class-specific counting benchmark focused exclusively on car instances. It consists of 1,448 drone-captured images from four different parking lots, with approximately 90,000 annotated car instances. The ShanghaiTech Part B dataset [50], commonly used for crowd counting, contains 716 images with a total of 88,488 annotated human instances.

#### Metrics.

In line with prior studies [15, 16, 21, 23, 24], we assess the counting method’s performance using Mean Absolute Error (MAE) and Root Mean Squared Error (RMSE), defined as follows:

$$MAE=\frac{1}{N_{t}}\sum_{i=1}^{N_{t}}\left|C_{i}-{\hat{C}}_{i}\right|$$ (9)

$$RMSE=\sqrt{\frac{1}{N_{t}}\sum_{i=1}^{N_{t}}{\left(C_{i}-{\hat{C}}_{i}\right)}^{2}}$$ (10)

where *N*_*t*_ is the number of test images, and *C*_*i*_ and ${\hat{C}}_{i}$ represent the ground-truth and predicted object counts for the *i*-th test image, respectively.

### Implementation details

We resized the image to 512 × 512, and applied data augmentation techniques including color jittering, horizontal flipping, and tiling. Tiling was applied to images, bounding boxes, and density maps. This augmentation technique divides the image into smaller small pieces (tiling), applies random transformations (e.g., horizontal flipping, scaling) to each piece, and then recombines them. This approach increases dataset diversity and enhances model generalization. The ground-truth density map for each image was generated using a Gaussian kernel function, with the kernel size set to $\frac{1}{8}$ of the average size of the exemplar bounding boxes.

In low-shot counting (LSC) methods, the classes in the training and test sets are different. To preserve generic semantic information learned from pre-trained models, the backbone is often initialized with weights trained on large datasets and then frozen (or partially frozen). Previous methods such as GMN [15], FamNet [16], and LOCA [24] use ResNet50 pre-trained on ImageNet [47] as the backbone, while CounTR [22] and CACViT [29] use a Vision Transformer (ViT) [38] pre-trained on ImageNet. In this paper, we employ SwinT-T [39] as the backbone, initializing its parameters with those of GroundingDINO [42], an open-vocabulary object detection model pre-trained on large-scale datasets. We trained our model using the AdamW optimizer [43] with a learning rate of 10^−4^ and weight decay of 10^−4^, running on an RTX A4000 GPU with a batch size of 4, completing training in under six hours.

### Comparison with state-of-the-art methods

Our model is evaluated on the FSC147 benchmark [16], following the standard evaluation protocols [15, 16, 21, 23, 24]. We compare the mean absolute error (MAE) and root mean squared error (RMSE) with other approaches.

#### Few-shot counting.

In the few-shot setting, each query image is accompanied by three support exemplars during both training and inference. Table 1 summarizes the results across different methods on the validation and test sets, including details on model backbone, image resolution, performance, and inference latency. To enhance generalization to novel object counting, we freeze the backbone in both training and testing phases. Although including small objects in high-resolution images aids accuracy, it also increases computational complexity—particularly for transformer-based methods. To balance performance and efficiency, we use a resolution of 512 for query images. As shown in Table 1, our model surpasses all others on the validation set and ranks second on the test set. Moreover, it achieves more than double the inference speed of the baseline LOCA [24], making it suitable for real-time applications. The CACViT [29] model, which has the best performance on the test set, aims to count objects in few-shots, unlike our model which can also perform zero-shot counting.

**Table 1:** Comparison with the state-of-the-art approaches on the FSC147 dataset under the few-shot counting scenario. The latency unit is milliseconds (ms).

Model	Year	Backbone	Resolution	FSC147-Val			FSC147-Test		
				MAE	RMSE	latency	MAE	RMSE	latency
GMN [15]	ACCV2018	ResNet-50	255	29.66	89.81	–	26.52	124.57	–
FamNet+ [16]	CVPR2021	ResNet-50	384	23.75	69.07	–	22.08	99.54	–
CFOCNet [17]	WACV2021	ResNet-50	256	21.19	61.41	–	22.10	112.71	–
BMNet+ [21]	CVPR2022	ResNet-50	384-1584	15.74	58.53	21.42	14.62	91.83	22.05
SAFECount [23]	WACV2023	ResNet-18	512	15.28	47.20	81.50	14.32	85.54	80.88
CounTR [22]	BMVC2022	ViT-B & CNN	384	13.13	49.83	89.59	11.95	91.23	79.85
CounTX [27]	BMVC2023	CLIP ViT&Text	224	17.10	65.61	46.67	15.88	106.29	41.30
CACViT [29]	AAAI2024	ViT-B & CNN	384	10.63	37.95	83.14	9.13	48.96	64.57
LOCA [24]	ICCV2023	ResNet50	512	10.24	32.56	44.77	10.79	56.97	45.37
Ours	–	Swin-T	512	9.27	30.09	18.22	10.11	55.79	17.92

#### One-shot counting.

In the one-shot scenario, only a single object annotation is provided. Table 2 presents a comparison of various methods, where our model outperforms others on the validation set and second best on the test set, while maintaining lower latency, demonstrating robustness even with limited data.

**Table 2:** Compare the evaluation results on a one-shot count scenario.

Model	FSC147-Val			FSC147-Test		
	MAE	RMSE	latency	MAE	RMSE	latency
CFOCNet [17]	27.82	71.99	–	28.6	123.96	–
FamNet+ [16]	26.55	77.01	–	26.76	110.95	–
BMNet+ [21]	17.89	61.12	–	16.89	96.65	–
LaoNet [44]	17.11	56.81	–	15.78	97.15	–
CounTR [22]	13.15	49.72	–	12.06	90.01	–
CACViT [29]	11.41	41.04	–	8.62	29.92	–
LOCA [24]	11.36	38.04	44.94	12.53	75.32	45.19
Ours	10.89	36.09	18.58	12.2	74.81	18.06

#### Zero-shot counting.

For zero-shot counting, where reference target annotations are missing, we used the zero-shot handling method from LOCA [24]. This method modifies the iterative module by removing Step 1 in Algorithm 1. Additionally, the embedding of $F_{exm}^{\prime k}$ in Step 2 is initialized using learnable object queries (${\mathbb{R}}^{s\times s\times d}$). For more details, please refer to LOCA [24]. As shown in Table 3, our model substantially outperforms all methods with a relative improvement of 29.0%, 11.5% in terms of MAE on validation and test sets, respectively, sets a solid new state-of-the-art.

**Table 3:** Compare with other methods in the zero-shot scenario.

Model	FSC147-Val			FSC147-Test		
	MAE	RMSE	latency	MAE	RMSE	latency
RepRPN-C [18]	29.24	98.11	–	26.66	129.11	–
RCC [19]	17.49	58.81	–	17.12	104.53	–
CounTR [22]	17.40	70.33	–	14.12	108.01	–
DAVE [30]	15.54	52.67	–	15.14	103.49	–
LOCA [24]	17.43	54.96	44.25	16.22	103.96	44.73
Ours	12.37	43.64	17.72	14.35	103.70	17.62

#### Qualitative analysis under various counting settings.

**Fig 7:**
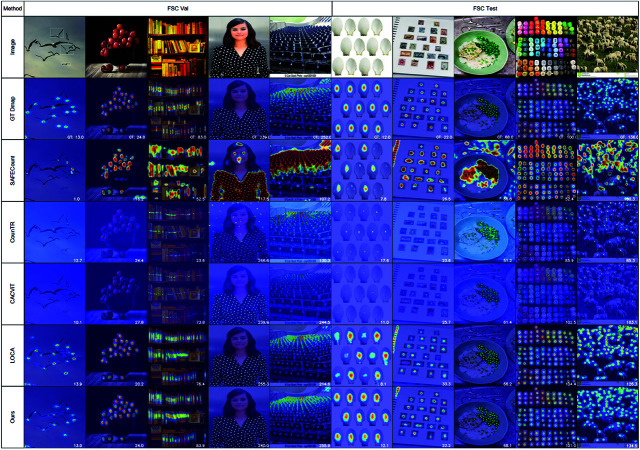
Qualitative results on the val and test set of FSC147 dataset. The selected images contain a variety of challenges. These include images with large variations in target size, target density and target depth.

To assess qualitative performance, we selected images with diverse target sizes and densities, overlaying predicted density maps onto original images for visualization. As illustrated in Fig 7, our method adapts effectively to different scenarios—including dense scenes, colorful environments, and large depth variations. These advantages demonstrate model’s ability to accurately localize and count objects.

### Cross-dataset generalization

#### Few-shot transfer to CARPK dataset with and without fine-Tuning.

Following established protocols in [16, 22–24, 29], we evaluated our model’s cross-dataset generalization by training on FSC147 dataset [16] and testing on CARPK dataset [8]. To focus on generalization, we excluded the ’car’ class from FSC147’s training set. In the few-shot scenario, twelve exemplars were sampled as supervised annotations.

For comparison, we conducted two evaluations: one without fine-tuning and one with fine-tuning. The results are presented in Table 4. Our model achieves better cross-dataset generalization under these two evaluations. From the penultimate row of the Table 4, it can be noticed that directly fine-tune the pre-trained model that without skip Car class, the model works on CARPK dataset. While fine-tuning with the pre-trained model that skip the Car class, our method outperforms the current state-of-the-art with a relative improvement of 22.8% MAE and 24.3% RMSE on the validation set.

**Table 4:** Compare the cross dataset generalization on CARPK under the settings of with and without fine-tuning.

Method	Year	Fine-tuned	MAE	RMSE
FamNet+ [16]	CVPR2021	×	28.84	44.47
RCAC [48]	ECCV2022	×	17.98	24.21
SAFECount [23]	WACV2023	×	16.66	24.08
BMNet+ [21]	CVPR2022	×	10.44	13.77
LOCA [24]	ICCV2023	×	9.97	12.51
CACViT [29]	AAAI 2024	×	8.30	11.18
Ours	-	×	8.03	9.59
FamNet+ [16]	CVPR2021	✓	18.19	33.66
RCAC [48]	ECCV2022	✓	13.62	19.08
BMNet+ [21]	CVPR2022	✓	5.76	7.83
CounTR [22]	BMVC2022	✓	5.75	7.45
SAFECount [23]	WACV2023	✓	5.33	7.04
LOCA [24]	ICCV2023	✓	5.14	6.50
CACViT [29]	AAAI 2024	✓	4.91	6.49
Ours(not skipCar)	-	✓	3.37	4.31
Ours(skipCar)	-	✓	3.79	4.91

#### Qualitative results on CARPK.

**Fig 8:**
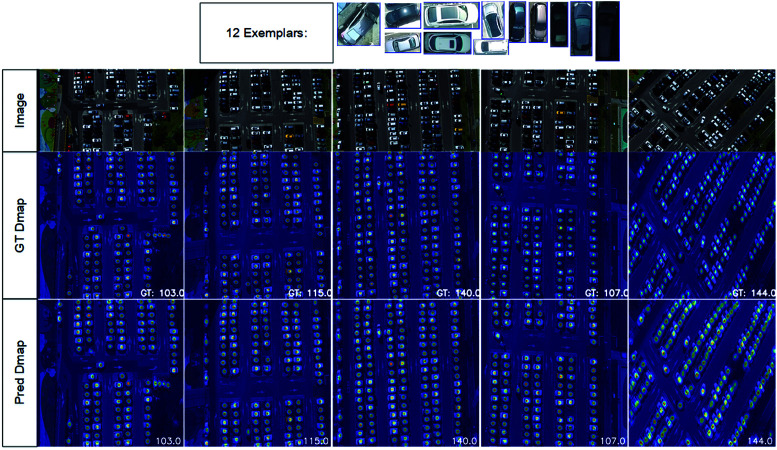
Visualize the results of crowded scenes from the CARPK dataset.

For CARPK, images originally sized 1280 × 720 were resized to 512×512 to reduce computational load. Fig 8 visualizes counting results, illustrating our model’s ability to accurately localize and count objects of various shapes, sizes, and densities.

#### Few-shot transfer to ShanghaiTech dataset.

We also evaluated our method on crowd counting datasets to assess its generalizability. Consistent with the settings in SAFECount, only five support images were randomly sampled from the training set and fixed for both fine-tuning and testing. The results are presented in Table 5. While the performance of our model is slightly lower, the number of epochs required for fine-tuning is significantly reduced (35 vs. 1000).

**Table 5:** Fine-tune the pretrained model on ‘ShanghaiTech PartB’ with few exemplars.

Methods	MAE	RMSE
FamNet	24.90	-
SAFECount	9.98	-
Ours	10.02	16.06

**Fig 9:**
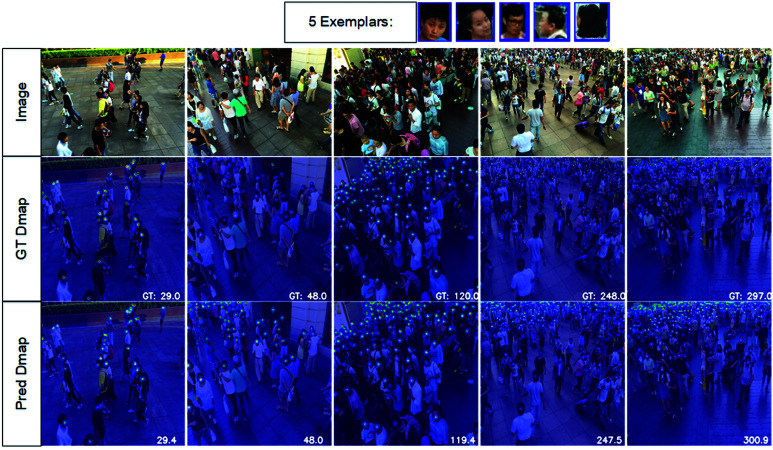
Qualitative results on the ShanghaiTech ParB dataset.

The qualitative results on ShanghaiTech are shown in Fig 9. The five given exemplars are shown on the top. We choose images containing different numbers of people for comparison. The positions of individuals are represented by dots, and the predicted total count is shown in the bottom-right corner.

#### Convergence speed during fine-tuning.

We compared performance using a pre-trained model with and without the car class. Table 6 shows that our model converges quickly, reaching optimal performance within four fine-tuning epochs.

**Table 6:** Fine-tuning on CARPK with the pre-trained model on FSC147. Skip Car means to remove the class ‘Car’ during pre-training.

epoch	Not Skip Car		Skip Car	
	MAE	RMSE	MAE	RMSE
1	7.02	8.51	7.34	8.76
2	4.35	5.52	9.55	10.96
3	3.45	4.50	3.81	4.93
4	3.37	4.31	3.79	4.91

### Comparison with object detectors on Val-COCO and Test-COCO

Val-COCO and Test-COCO [16] are FSC-147 subsets derived from COCO, designed for evaluating object counting models. We compared our model against several detection-based models, including Faster-RCNN [45], RetinaNet [46], Mask-RCNN [41], and recent few-shot models such as FamNet [16], BMNet+ [21], CounTR [22], and LOCA [24]. Results for direct evaluation on Val-COCO and Test-COCO (without fine-tuning) using our pre-trained model are shown in Table 7, from which we find that our model gets better performance.

**Table 7:** Comparison of model generalizability on Val-COCO and Test-COCO. Validation was performed directly with the pre-trained weights on FSC147 without fine-tuning.

Method	Val-COCO		Test-COCO	
	MAE	RMSE	MAE	RMSE
Faster-RCNN [45]	52.79	172.46	36.20	79.59
RetinaNet [46]	63.57	174.36	52.67	85.86
Mask-RCNN [41]	52.51	172.21	35.56	80.00
Famnet [16]	39.82	108.13	22.76	45.92
BMNet+ [21]	26.55	93.63	12.38	24.76
CounTR [22]	24.66	83.84	10.89	31.11
LOCA [24]	16.86	53.22	10.73	31.31
Ours	15.31	43.56	8.09	15.93

### Ablation study

#### Ablation on the adaptive generation of ground truth density map.

We propose a dynamic adjustment of $\bar{h}$ and $\bar{w}$ based on the inter-point distances in high-density regions, thereby generating improved density maps. The steps are shown in the Algorithm 2. The threshold α=0.3 and the weight parameter 1.6 in $\beta_{2}$ are set based on the experiments. We conducted experiments for α within the range of [0.2, 0.5] with an interval of 0.05, and for the weight parameter within the range of [1, 2] with an interval of 0.1. The optimal values for both parameters were selected based on the results. These ablation experiments were all conducted on the FSC147 dataset under the few-shot setting.

We compare the results before and after applying the dynamic adjustment of density maps in Table 8. From the table, it can be observed that the dynamically generated density maps improved the model’s performance on both the validation and test sets, demonstrating the effectiveness of the module.

**Table 8:** Comparison of the results between the model with and without the dynamic density map generation method.

Method	Val		Test	
	MAE	RMSE	MAE	RMSE
without	9.56	34.78	10.51	70.64
with	9.27	30.09	10.11	55.79

To further validate the method, we refer to the CACViT [29] method, dividing images into subsets based on the number of targets they contain. The low-density subset includes images with 8–37 targets, while the high-density subset contains 37–3701 targets. The experimental results are shown in Table 9.

**Algorithm 2. Dynamic adjustment of Gaussian parameters for density map generation.**

Input:

  The distribution map of target center points *P* in the image.

  The average height and width of the given *K*-shot annotated boxes: $\bar{h}$ and $\bar{w}$

  The distance metric of all points in the image *d*.

Output:

  Dynamically adjusted density map $D_{\text{map}}$.

Steps:

  1. Compute the global mean distance $d_{\text{mean}}=\frac{1}{n}\sum_{i=1}^{n}d_{i}$.

  2. Roughly select points that may belong to high-density regions using the condition: $0.7\times d_{\text{mean}}>d_{i}$

  3. Classify the image into high-density and low-density regions based on the Eq (1).

  4. Generate Density Map for Low density Regions using the Eq (2), where $\beta_{1}=\frac{1}{8}$.

  5. Generate Density Map for High-Density Regions: a) Calculate the average points distance that belongs to high density regions $d_{\text{den}}$. b) Dynamically adjust the Gaussian gamma parameter using the formula: $\beta_{2}=\frac{d_{\text{den}}}{d_{\text{mean}}}\times 1.6\times \beta_{1}$, where $\beta_{2}=1$ if $\beta_{2}>=1$. c) Apply the adjusted gamma to generate the high density map using Eq (3).

  6. Combine low density and high desity map to obtain the final density map $D_{\text{map}}$.

**Table 9:** Comparison of performance on low-density and high-density subsets.

Method	Val Low		Val High		Test Low		Test High	
	MAE	RMSE	MAE	RMSE	MAE	RMSE	MAE	RMSE
BMNet+[21]	4.74	7.56	32.3	92.2	6.23	29.96	23.21	127.62
CounTR[22]	4.25	8.54	26.34	76.63	5.44	36.96	18.56	124.11
CACViT[29]	3.53	6.85	21.33	59.50	3.14	6.26	15.33	69.54
LOCA[24]	4.31	7.73	19.13	50.60	5.02	20.36	15.11	68.63
Ours	3.59	6.23	16.82	47.02	4.80	9.54	13.51	68.17

#### Encoder ablation.

**Fig 10:**
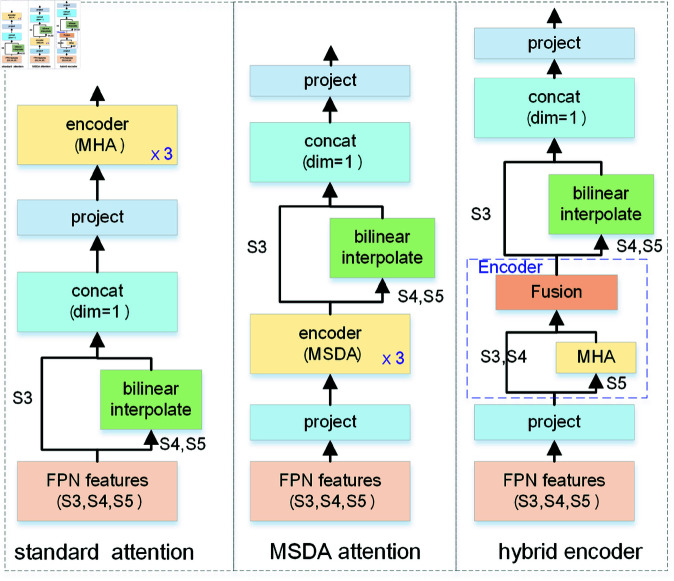
Comparative structures of different attention mechanisms in encoders.

To evaluate the effectiveness of our hybrid encoder, we experimented with three encoder structures: one using standard multihead attention (MHA), another using multi-scale deformable attention (MSDA), and the proposed encoder. These module structures are illustrated in Fig 10.

We conduct experiments on the FSC147 dataset under few-shot settings to compare the performance of various encoders. Each input image is resized to 512×512 pixels. High-level features are extracted from the backbone at different stages: $S_{3}\in {\mathbb{R}}^{64\times 64\times 192}$, $S_{4}\in {\mathbb{R}}^{32\times 32\times 384}$, and $S_{5}\in {\mathbb{R}}^{16\times 16\times 768}$. Given an input image, the processing pipeline is as follows:

(a) Standard transformer attention (3 layers): $S_{3},S_{4},S_{5}\overset{interpolate}{\to }S_{3}\in {\mathbb{R}}^{64\times 64\times 192},S_{4}\in {\mathbb{R}}^{64\times 64\times 384},S_{5}\in {\mathbb{R}}^{64\times 64\times 768}\overset{concat}{\to }{\mathbb{R}}^{64\times 64\times 1344}\overset{project}{\to }{\mathbb{R}}^{64\times 64\times 256}\overset{MHA\;encoder}{\to }{\mathbb{R}}^{64\times 64\times 256}$

(b) Multi-scale deformable attention (3 layers): $S_{3},S_{4},S_{5}\overset{project}{\to }S_{3}\in {\mathbb{R}}^{64\times 64\times 256},S_{4}\in {\mathbb{R}}^{32\times 32\times 256},S_{5}\in {\mathbb{R}}^{16\times 16\times 256},\overset{MSDA\;encoder}{\to }S_{3}\in {\mathbb{R}}^{64\times 64\times 256},S_{4}\in {\mathbb{R}}^{32\times 32\times 256}$, $S_{5}\in {\mathbb{R}}^{16\times 16\times 256}\overset{interpolate}{\to }S_{3}\in {\mathbb{R}}^{64\times 64\times 256},S_{4}\in {\mathbb{R}}^{64\times 64\times 256},S_{5}\in {\mathbb{R}}^{64\times 64\times 256}\overset{concat}{\to }{\mathbb{R}}^{64\times 64\times 768}$ $\overset{project}{\to }{\mathbb{R}}^{64\times 64\times 256}$

(c) Hybrid encoder (1 layer): $S_{3},S_{4},S_{5}\overset{project}{\to }S_{3}\in {\mathbb{R}}^{64\times 64\times 256},S_{4}\in {\mathbb{R}}^{32\times 32\times 256},S_{5}\in {\mathbb{R}}^{16\times 16\times 256}\overset{hybrid\;encoder}{\to }S_{3}\in {\mathbb{R}}^{64\times 64\times 256},S_{4}\in {\mathbb{R}}^{32\times 32\times 256},S_{5}\in {\mathbb{R}}^{16\times 16\times 256}\overset{interpolate}{\to }S_{3}\in {\mathbb{R}}^{64\times 64\times 256},S_{4}\in {\mathbb{R}}^{64\times 64\times 256},S_{5}\in {\mathbb{R}}^{64\times 64\times 256}\overset{concat}{\to }{\mathbb{R}}^{64\times 64\times 768}\overset{project}{\to }{\mathbb{R}}^{64\times 64\times 256}$

We evaluated three encoders on the FSC147 validation set, comparing their MAE, parameter numbers, and latency. The results are summarized in Table 10. Although our hybrid encoder includes a CNN-based fusion module that slightly increases the parameter numbers, it achieves low latency without compromising performance.

**Table 10:** Comparison of model performance and latency across different encoders.

Method	Backbone	Val MAE	Parameters (M)		Latency(ms)
			Total	Trainable	
BMNet+[21]	ResNet-50	15.74	13.08	12.86	21.42
SafeCount[23]	ResNet-18	15.28	32.11	20.42	81.50
LOCA[24]	ResNet50	10.24	36.88	11.37	44.77
CounTR[22]	ViT-B & CNN	13.13	99.69	98.95	65.00
CACViT[29]	ViT-B & CNN	10.63	100.53	99.77	110.07
Swin-MHA	SwinT-B	10.16	38.32	10.08	20.08
Swin-MSDA	SwinT-B	9.93	37.02	10.89	24.01
Swin-hybrid	SwinT-B	9.27	44.55	18.43	18.22

We also tracked on the size of GPU and memory required by LOCA and our method, while training on FSC147 in few-shot settings. As shown in the Table 11, which shows that for the same training setup, our model requires considerably less GPU memory and a decrease in CPU memory.

**Table 11:** Comparison on GPU and memory size during training.

Method	Shots	GPU (G)	Memory (G)
LOCA	3-shot	29.11	10.14
	1-shot	28.94	7.97
	0-shot	29.05	7.49
Ours	3-shot	3.22	7.20
	1-shot	2.90	6.26
	0-shot	3.19	5.31

#### Ablation study on the exemplar feature learning module.

**Fig 11:**
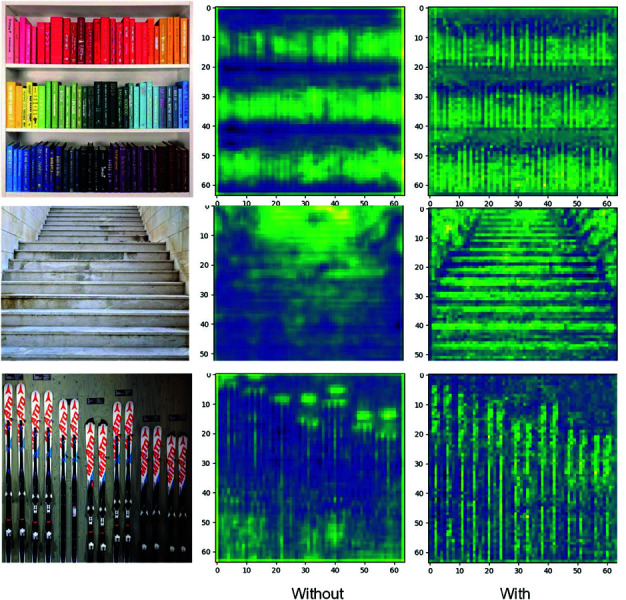
Visualization of response maps with and without the exemplar feature learning module.

To evaluate the effectiveness of the i-EFL module, we analyzed the response maps generated with and without it. After matching exemplar features with image features, the model produces a response map, denoted as $R_{map}\in {\mathbb{R}}^{64\times 64\times 256}$. For visual comparison, we project this response map to $R_{map}\in {\mathbb{R}}^{64\times 64\times 1}$. As shown in Fig 11, the features are more effectively learned with the enhanced exemplar feature.

#### Comparison of results across different categories.

**Fig 12:**
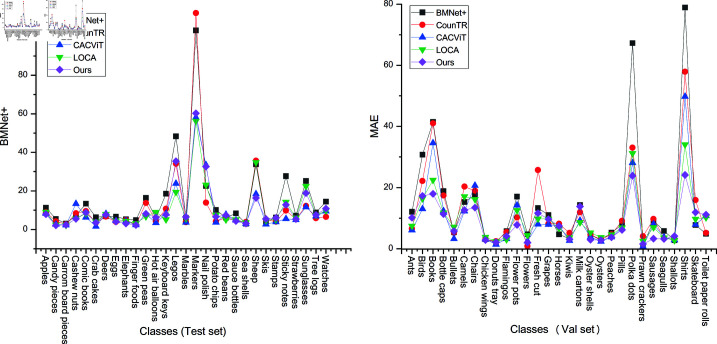
Comparison the average MAE for each class.

As shown in Fig 6, the distribution of the number of objects across categories in the FSC147 dataset is highly imbalanced. We compared and visualized the average MAE for each category in the validation and test sets, with the results presented in Fig 12. From the figure, we can observe that the performance has slightly improved for categories with highly imbalanced densities, such as *books*, *chairs* and *shirts*.

#### Analysis of the auxiliary loss weight setting.

The auxiliary loss is defined as the sum of two intermediate layer errors. To balance it with the primary loss, ${ℒ}_{C}$, we conducted experiments using λ values ranging from 0 to 0.5 in increments of 0.1. These experiments were carried out on the FSC147 dataset under few-shot learning scenarios. The results, summarized in Table 12, demonstrate that incorporating auxiliary loss enhances the model’s performance. However, an excessive auxiliary loss can negatively impact the optimization of the primary task.

**Table 12:** Analysis of the impact of auxiliary loss weight on model performance.

lambada	0.0	0.10	0.20	0.30	0.40	0.50
Val MAE	9.76	9.42	9.40	9.27	9.35	9.54

#### Convergence speed and performance comparison.

**Fig 13:**
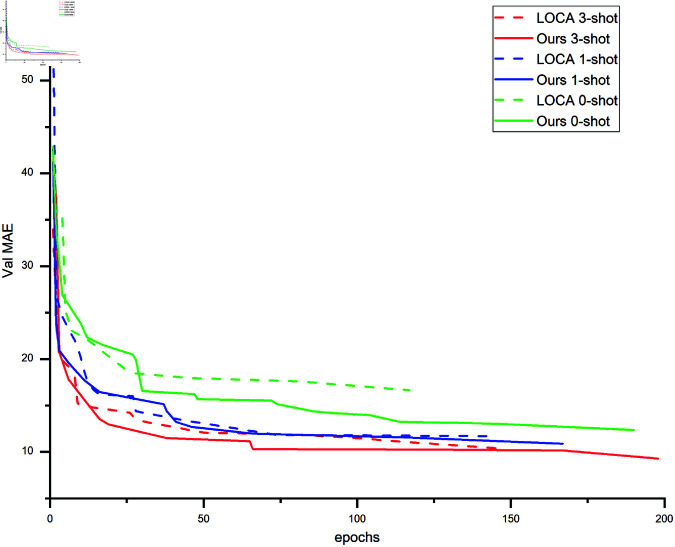
Comparison of MAE and convergence speed with LOCA.

LOCA [24] represents the state-of-the-art in density-based methods. We compare our model’s convergence speed and performance during training under different few-shot settings. As shown in Fig 13, our model achieves faster convergence and improved performance across various settings.

**Fig 14:**
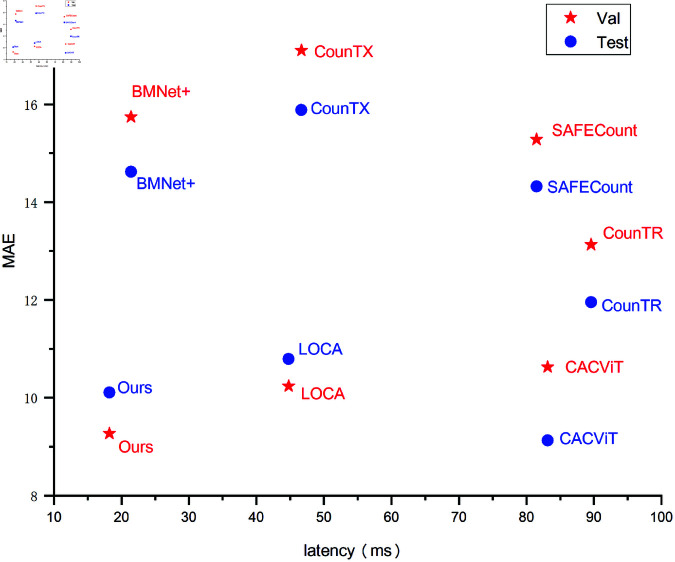
Comparison of MAE and latency.

We compare the MAE and latency across different methods, as shown in Fig 14. Our model achieves the best performance and lowest latency on the validation set. On the test set, although our model’s performance ranks second to CACViT, it demonstrates significantly lower latency (17.93 ms vs. 64.57 ms).

## Discussion

The application of AI technology for analyzing and processing images holds substantial potential for enhancing data-driven decision-making across various domains. This work addresses the generalized visual object counting problem, specifically counting objects from arbitrary categories using only a few exemplars. To balance the performance and inference speed, we design a hybrid encoder module that reduces model complexity and an iterative exemplar feature enhancer module that boosts performance. Additionally, we employ a synthetic approach to generate more accurate ground-truth density maps. Experiments on FSC-147, Val-COCO and Test-COCO demonstrate that our method meets real-time requirements while achieving high accuracy, experiments on CARPK and ShanghaiTech demonstrate model’s generalizability.

## Supporting information

S1 Fig Different domain images in FSC147 dataset. (TIF)

S2 Fig Application in different domain results. (TIF)
