# Supplementary figures and images for: An efficient low-shot class-agnostic counting framework with hybrid encoder and iterative exemplar feature learning
Source: PLoS One. 2025 Jun 6;20(6):e0322360. doi: 10.1371/journal.pone.0322360 (PMC12143539; doi:10.1371/journal.pone.0322360)

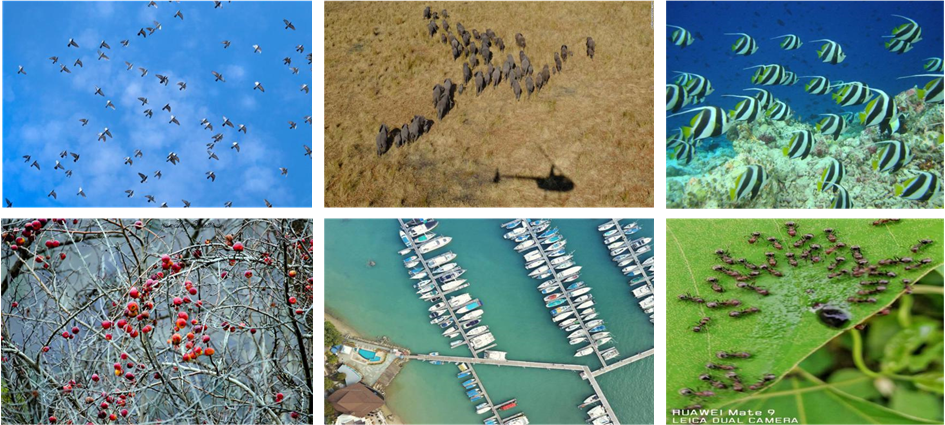

Supplement: S1 Fig — (TIF) [file pone.0322360.s001.tif]

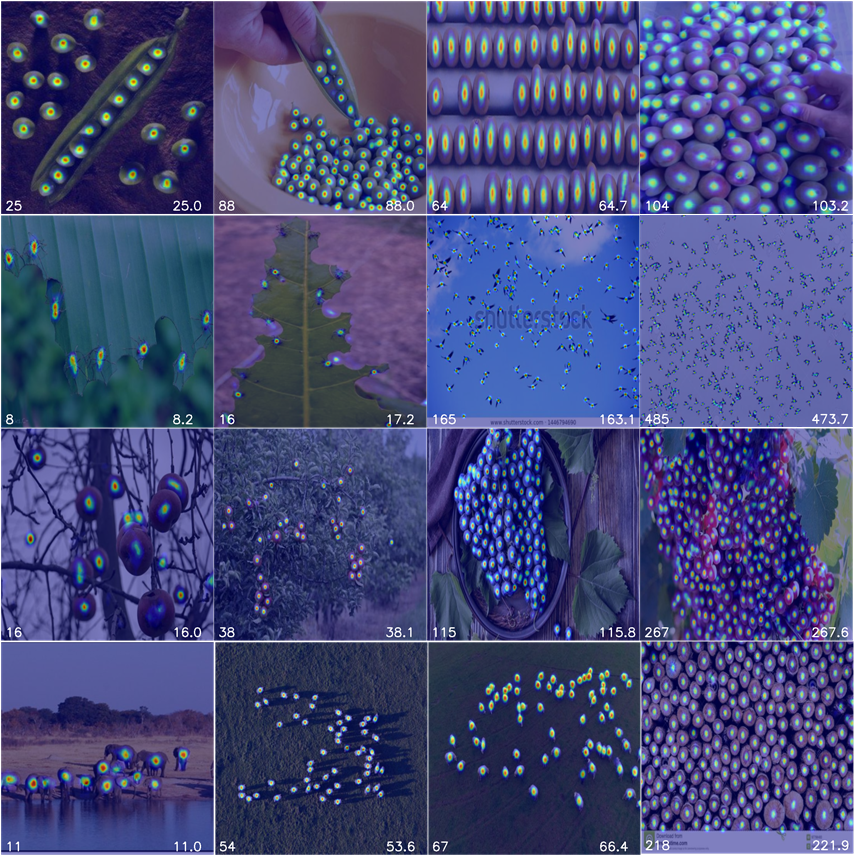

Supplement: S2 Fig — (TIF) [file pone.0322360.s002.tif]
